# Successful management of an advanced interstitial ectopic pregnancy in a resource-limited setting: a case report

**DOI:** 10.1186/s13256-024-04437-y

**Published:** 2024-03-20

**Authors:** Clovis Achassi Tankeng, Quinta Mua Ekei, Yannick Lechedem Ngunyi, Eugene Vernyuy Yeika, Elvis Nkengasong Ajabmoh, Alfred Awa Mokom

**Affiliations:** 1 Faculty of Health Sciences, University of Buea, Buea, Cameroon; 2 Our Lady of Lourdes Medicalized Health Center, Nkar-Jakiri, Cameroon; 3 Mbonge District Hospital, Mbonge, Cameroon; 4 Hopital Saint Therese de l’enfant Jesus de Nkolbisson, Yaounde, Cameroon; 5 Kahwa Sumbele Medical Clinic, Buea, Cameroon

**Keywords:** Advanced interstitial ectopic pregnancy, Laparotomy, Case presentation, Cameroon

## Abstract

**Background:**

Interstitial ectopic pregnancy is an ectopic gestation developing in the uterine part of the fallopian tube. The condition is rare and presents challenges for clinical as well as radiological diagnosis. This case report presents a rare case of interstitial ectopic pregnancy diagnosed intraoperatively.

**Case presentation:**

A 36-year-old Black woman, referred from a peripheral health facility, presented at the emergency department with severe abdominal pains, vaginal spotting, nausea, and vomiting, with a 2-month history of irregular menstrual flow. Clinical and laboratory findings were suggestive of an acute abdomen likely due to a ruptured ectopic pregnancy (ultrasound was not available). An emergency exploratory laparotomy was done, which revealed a right adnexal ruptured interstitial pregnancy of a lifeless female fetus weighing 500 g (estimated mean gestational age of 22–23 weeks). The left fallopian tube looked normal. The site of rupture was repaired, followed by cleaning and closure of the abdomen. The post-operative period was uneventful, and the patient was discharged on postoperative day 7.

**Conclusion:**

Interstitial pregnancies are uncommon and rarely attain advanced gestational ages, as in this case, compared with other tubal ectopic pregnancies. However, women presenting with signs of hypovolemic shock and acute abdomen, with a positive pregnancy test, warrant a high index of suspicion.

## Background

**Fig. 1:**
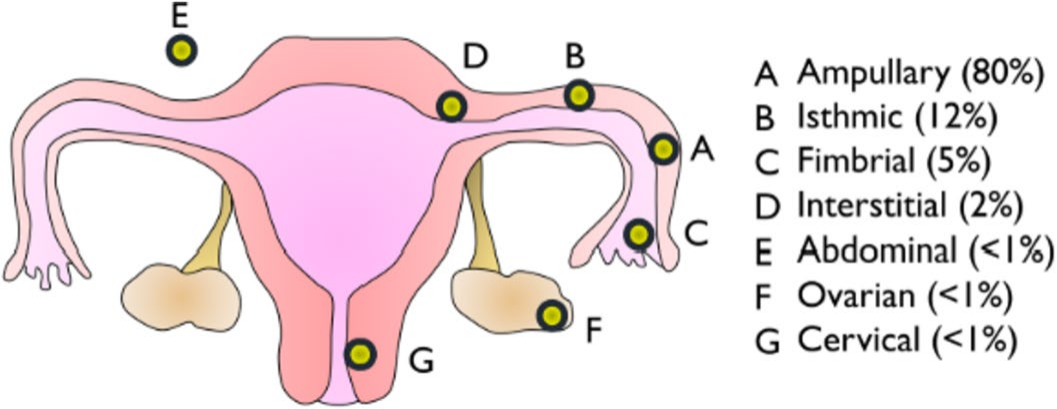
Location and prevalence of ectopic pregnancies [4]

An ectopic pregnancy occurs in 1–2% of pregnancies and accounts for 6–9% of pregnancy-related deaths [1–3]. It is the leading cause of maternal mortality in the first trimester of pregnancy. Non-tubal ectopic pregnancies, including interstitial pregnancies, are rare and account for 5–10% of ectopic pregnancies (Fig. 1) [2, 4, 5]. Interstitial ectopic pregnancy comprises 1–11% of all ectopic pregnancies. It has been reported in the first trimester to early second trimester, presenting relatively later compared with other tubal pregnancies owing to the overlying myometrial muscles that give it the ability to expand and accommodate larger fetuses [6]. It is common to present in rupture or impending rupture as other ectopic pregnancies owing to the diagnostic challenges, which are both clinical and related to imaging, including non-specific clinical signs, symptoms, and ultrasonographic signs [4, 7].

Interstitial ectopic pregnancy presents similarly to other tubal ectopic pregnancies with symptoms such as abdominal pain, spotted vaginal bleeding, nausea, light-headedness, and asthenia. However, the severity of symptoms depends on whether it is ruptured or not, the degree of bleeding, and the hemodynamic status [4, 6]. Ultrasonography remains the gold standard of diagnosis, where an empty uterus and an interstitial line or sac is highly suggestive [4, 7]. Most interstitial ectopic pregnancies are usually diagnosed during laparotomy as ruptured ectopic pregnancy [8]. As with other ectopic pregnancies, its risk factors include pelvic inflammatory disease, artificial reproductive therapy, smoking, use of progesterone contraceptive pills, and history of ectopic pregnancy. The management of interstitial ectopic pregnancy can be conservative, medical, or surgical depending on the size of the sac/embryo, presentation, level of B-HCG, ultrasound finding, hemodynamic status of the patient, and evolution with time [4]. Most women present with rupture or impending rupture and hemodynamic compromise, which necessitates surgical management [2, 8]. Surgery remains the treatment of choice when there is rupture, impending rupture, and/or hemodynamic compromise [5, 8–10].

Data reporting interstitial ectopic pregnancy at gestational age greater than or equal to 20 weeks’ gestation are rare. In this case report, we present a case of a developed interstitial ectopic pregnancy found during laparotomy for ruptured ectopic pregnancy on a 36-year-old woman referred to our setting in a state of hemodynamic instability after a sudden collapse. Reporting this case, which is quite rare and managed in a rather resource-limited setting, enriches the literature.

## Case presentation

We present a 36-year-old Black female gravida 4, para 3 (G_4_P_3_; three previous pregnancies, three term deliveries, with two live children; she lost her first son at the age of 2 years), with a 2-month history of irregular menses. She was referred to our facility with complaints of sudden severe abdominal pain associated with vomiting and generalized body weakness. There was also fatigue, headache, and spotted vaginal bleeding.

She has a regular cycle of 28 days, bleeds for 3 days, and uses two to three moderately soaked pads per day, but noticed a change in her last two previous menstrual flows. In August her flow lasted 1 day, and she used one pad, while in September it was 2 days, and she also used one pad. There was no history of dysmenorrhea, metrorrhagia, or dyspareunia. There was no prior history of ectopic pregnancy. She had never been treated for sexually transmitted diseases or pelvic inflammatory disease, and her mode of contraception is calendar. She had never been operated on and never been diagnosed with a chronic disease. She did not smoke but consumed alcohol occasionally.

Physical examination on admission revealed a conscious patient in an antalgic position. She was in shock [blood pressure (BP) 80/50 mmhg and pulse 138 beats per minute] and pale. The abdomen was distended, and there were signs of peritoneal irritation. A vaginal speculum exam revealed spotted bleeding from the cervical os, and a diagnosis of acute abdomen was made. Two large bore intravenous cannulae were placed, and blood was collected for a pregnancy test, hemoglobin, grouping, and cross-match. The patient was then resuscitated with crystalloids. The serum pregnancy test was positive, and hemoglobin was 7.7 g/dL. The working diagnosis of ruptured ectopic pregnancy was then made.

Ultrasonography was unavailable, and the possibility of referring her for one was not feasible, considering the emergency involved. The theater and anesthetic team were called immediately for an emergency laparotomy.

**Fig. 2:**
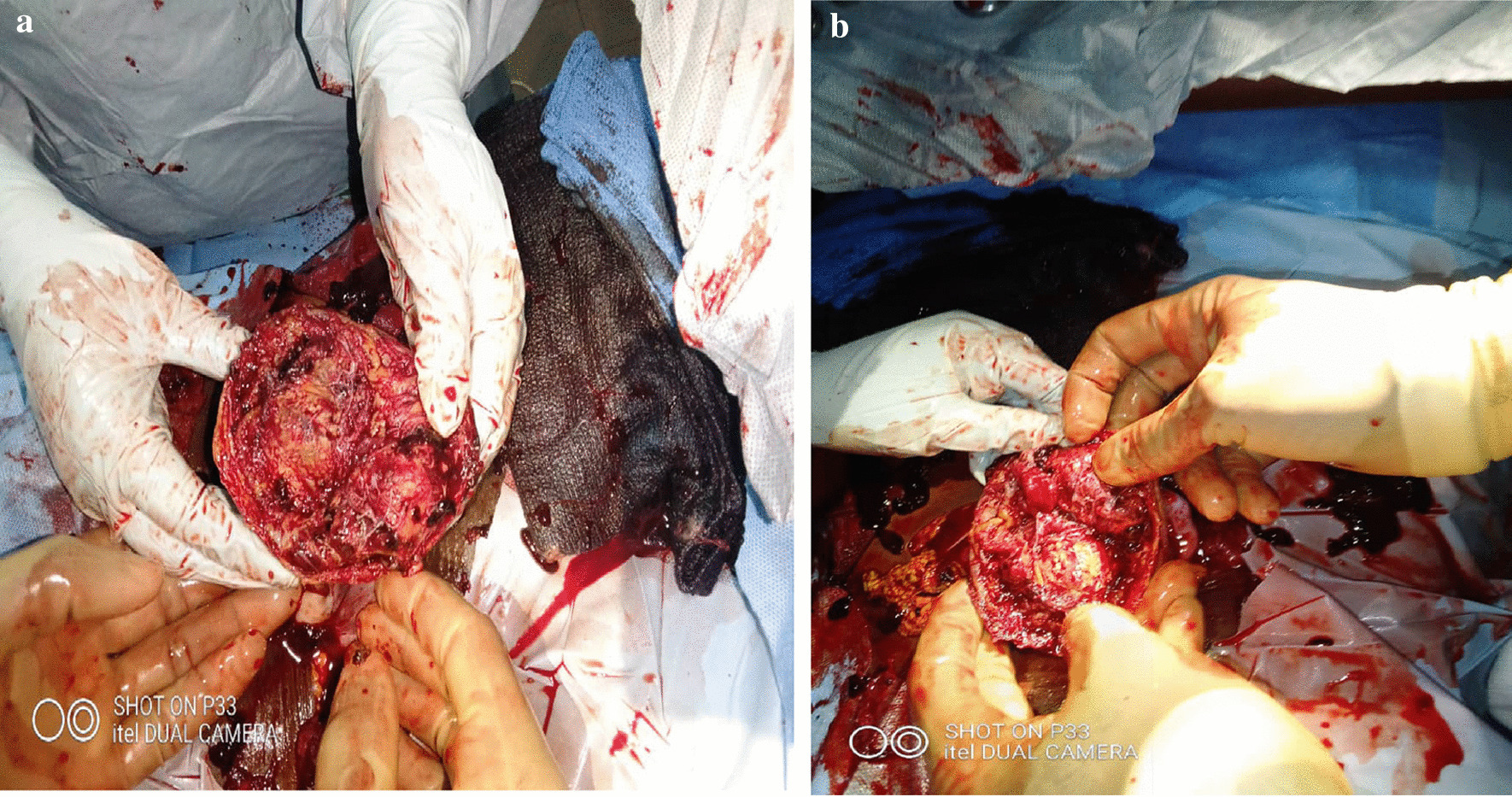
**a, b** Rupture site of the ectopic pregnancy at the utero-tubal junction but not communicating with endometrium

Intraoperatively, there was a massive hemoperitoneum. A well-formed female fetus weighing 500 g was found floating with a placenta (800 g) attached close to the left fundal tubo-uterine junction (deep to the serosa) with no sign of life, nor maceration. The site of rupture was at the upper left interstitial portion of the tube (Fig. 2a, b). A betadine–saline solution injected through the cervical os was seen dropping through the infundibulum of the tubes (both sides) but showed no communication with the ruptured site. The tubes were therefore of normal anatomy and patent with no signs of inflammation. The ruptured site was closed, the abdomen cleaned, and lavage done with lukewarm normal saline and closed. Blood loss was estimated at about 2.5 L.

The patient received 2 units of whole blood and an additional 2 L of crystalloids intraoperatively.

The operation was aseptic. However, she was placed on intravenous antibiotic prophylaxis, analgesics, fluids, and nil by mouth for 24 hours. She also received a third and fourth unit of whole blood on postoperative days 1 and 2, respectively. The progress in the ward was favorable, as all the symptoms subsided, and vitals stabilized on postoperative day 1. She ambulated on the second day and was relayed on oral antibiotics and analgesic. Control hemoglobin on day 3 was 8.7 g/dL, and she was placed on oral iron and folic acid at therapeutic doses.

She was then discharged on postoperative day 7 after education on menstrual health and counseling on family planning and the risk on subsequent pregnancies with a follow-up in 6 weeks.

## Discussion and conclusion

In our case, intraoperatively, the placenta was found attached close to the utero-fundal junction with no signs of communication with the uterine cavity. However, the tubes were patent, as the injected sterile solution was seen dripping through the infundibula of the tubes. The fetus weighed 500 g, about 800 g with placenta, with an estimated gestational age of at least 22 weeks [11]. The estimated gestational age from the mother’s last menstrual flow (9 weeks and 4 days) did not correlate with the size of the fetus, suggesting the patient’s poor knowledge of menstrual periods. Interstitial ectopic pregnancy has rarely been reported to attain gestational ages of more than 20 weeks. However, compared with other ectopic pregnancy types, it can possibly attain a higher gestational age owing to the overlying myometrium, which gives it an ability to distend [6]. This patient presented with signs and symptoms suggestive of an acute abdomen. The result of the pregnancy test and the state of hypovolemic shock made a ruptured ectopic pregnancy most likely, although they were not specific in regard to the site of implantation. The management of this case had several challenges—firstly, the sociopolitical crisis that has made transportation of patients difficult and risky. There were no ambulances, and the only means of transport were a few bikes, which increased the risk of death given the hemodynamic instability of the patient, and the worsening prognosis with time. Owing to the poor prognosis and further risk of death with transportation, time, the crisis situation, and the distance to the next hospital, the decision for an urgent laparotomy was made, as it was the best available option to save the patient’s life.

Interstitial ectopic pregnancies are rare and constitute only 1–2% of ectopic pregnancies. They have been reported during late first trimester or early second trimester [6]. It is rare to find data on tubal ectopic pregnancies at mid-to-late second trimester, as in the case above. This supports the idea that interstitial pregnancies might be able to attain unexpectedly advanced gestational ages, as explained by Brincat *et al*. in a review study [6].

Many implantation sites for this case could be argued. It could have been an abdominal pregnancy whose placenta implanted on the fundus of the uterus, but this was less likely because, intraoperatively, there was a flap of uterine serosa over the site of rupture that approximately apposed each other during suturing. Another possible explanation was that it could have been an intrauterine pregnancy that implanted deep into the myometrium (from a cornual or angular implantation), and then protruded into the abdominal cavity. This was also less likely because the utero-tubal junction was intact and the rupture lateral to it. More so, there was no communication between the site of rupture and the intra-uterine cavity, which would have supported this fact [3]. The interstitial portion of the tube is covered by a layer of myometrium, which might give it some ability to expand and accommodate a larger fetus compared with tubal ectopic pregnancies [6]. This probably explains why this baby could have attained such a size with no symptoms until a few days prior to consultation.

Early detection of ectopic pregnancies through screening of suspected sexually active females with the use of transvaginal ultrasound and serial B-HCG measurements is an important step toward management of ectopic pregnancies. These are unavailable in our context, coupled with the current sociopolitical crisis, which has greatly affected the movement of patients to hospitals [6, 8]. On the part of the women, most are less educated on women’s health and menstrual cycles, and thus are unaware of their pregnancy state until signs and symptoms of rupture or impending rupture, as in the case of our patient [2, 6]. Being less educated on their menstrual cycle means they will not notice changes in their menstrual cycle that could prompt them to consult on time [4].

Interstitial ectopic pregnancy remains a rare type of ectopic pregnancy that is rarely reported, let alone extends beyond early second trimester.

The case presentation above, therefore, reinforces the idea that interstitial pregnancies may occasionally evolve to advanced gestational ages. It is important to educate women on their menstrual health, as this will enable cases of ectopic pregnancies to be reported earlier, reducing the risk of adverse maternal outcomes. This is especially true for this area, which is highly affected by the sociopolitical crisis that has reduced patient accessibility to healthcare. As such, a high index of suspicion is necessary for women presenting with signs of hypovolemic shock, an acute abdomen, and a positive pregnancy test. Also, in the absence of reliable ultrasonography, a lifesaving laparotomy should not be delayed, even in resource-limited settings, if referral to an advanced center is not possible within minutes.

## Data Availability

The data sets are included within the article.

## Abbreviations

B-HCG: Beta-human chorionic gonadotropin
IEP: Interstitial ectopic pregnancy
PID: Pelvic inflammatory disease
